# Mechanistic targets for the ablation of atrial fibrillation

**DOI:** 10.21542/gcsp.2017.7

**Published:** 2017-03-31

**Authors:** Junaid A.B. Zaman, Tina Baykaner, Amir A. Schricker, David E. Krummen, Sanjiv M. Narayan

**Affiliations:** 1 Stanford University, Palo Alto, California; 2 Imperial College, University of London, United Kingdom; 3 University of California, San Diego, California

## Abstract

The mechanisms responsible for sustaining atrial fibrillation are a key debate in cardiovascular pathophysiology, and directly influence the approach to therapy including ablation Clinical and basic studies have split AF mechanisms into two basic camps: ‘spatially distributed disorganization’ and ‘localized sources’. Recent data suggest that these mechanisms can also be separated by the method for mapping – with nearly all traditional electrogram analyses showing spatially distributed disorganization and nearly all optical mapping studies showing localized sources We will review this dichotomy in light of these recently identified differences in mapping, and in the context of recent clinical studies in which localized ablation has been shown to impact AF, also lending support to the localized source hypothesis. We will conclude with other concepts on mechanism-based ablation and areas of ongoing research that must be addressed to continue improving our knowledge and treatment of AF.

## 1. Introduction

Atrial fibrillation (AF) affects 30 million people globally^1^ making it the commonest sustained heart rhythm disorder It is also a major cause of hospitalizations, stroke and death. Therapy is based on drugs or ablation, yet both remain suboptimal due to uncertain mechanisms and drug side-effects

A fundamental mechanistic dichotomy in human atrial (and ventricular) fibrillation is whether disorder is driven by self-sustaining disorganization or by discrete organized processes. Although little overlap exists between each camp, it is increasingly apparent that they may reflect the mapping technique used rather than experimental model of AF or underlying pathophysiology Studies using optical mapping of action potentials, that indicate activation and recovery, mostly show localized sources that drive disorganized activation in fibrillation,^2,3^ yet studies using clinical electrograms (surrogates for cellular activation) mostly do not. Understanding how these techniques differ in representing cellular activity in fibrillation may help to advance this crucial mechanistic debate.^4^

This review first briefly outlines the mechanism of fibrillation in animal models described by different mapping techniques.^2^ Given the paucity of optical mapping studies of cardiac fibrillation in humans, we will use clinical mapping results to compare and contrast clinical results and discuss mechanistic implications We then highlight clinical data with outcomes of AF ablation that promise patient tailored therapy.

## 2. Mapping Approaches to Atrial Fibrillation

To map fibrillation one must identify atrial activation and repolarization at high temporal and spatial resolution without signal contamination (crosstalk) from adjacent sites. In organized rhythms such as atrial flutter, where all regions activate 1:1, crosstalk may alter activation time at each electrode but overall has minimal importance and so most mapping techniques agree. However, in fibrillation, adjacent tissue regions may reflect unrelated (dissociated) activation at different rates Thus, in fibrillation, crosstalk may contaminate local signals with dissociated far-field detections that may augment, cancel or modify activity and dramatically change mapping. Repolarization information available from optical mapping, monophasic action potentials and other techniques provide data to identify whether given deflections lie within tissue refractoriness (i.e., are far field) or are local.

**Figure 1.**
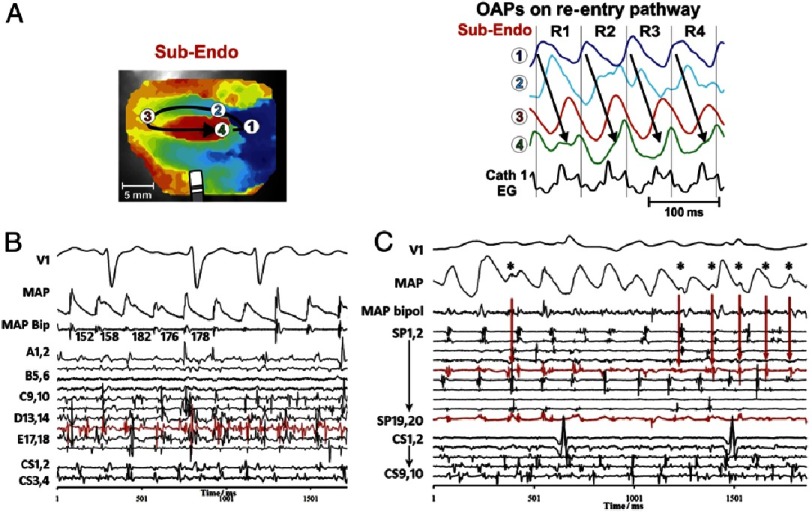
Optical mapping addresses limitations of electrogram mapping. A) Sub-endocardial optical mapping of human atria shows a stable rotor driving AF. Optical action potentials (OAPs) at sites 1–4 show activation and recovery over multiple sequential cycles. Note the complex fractionated electrograms (Cath 1 EG) despite the organized action potential signals. From [6]. B) Human AF showing clear and discrete monophasic action potentials on (MAP) catheter yet very complex bipolar electrograms representing far-field wavelet collision. C) Clinical bipoles reveal signals indistinguishable from local activation that actually represents far-field activation on the MAP. Signals in panels B, C greatly complicate AF maps from bipolar signals. From [11].

Optical mapping of voltage-sensitive dyes is often considered the gold standard to map complex arrhythmias.^5^ In this technique, light excites voltage sensitive dyes that fluoresce in proportion to membrane potential Each map shows the optical action potential (hence activation and recovery) at multiple sites and successive images represent propagation of the wavefront Optical mapping has recently been used to map AF in human atria.^6^ Crucially, optical maps show minimal signal crosstalk – essentially within the small area optically mapped by each image pixel. There has long been a call to apply optical imaging to human AF in order to categorically identify mechanisms.^5^ Unfortunately, optical mapping is not feasible in patients *in vivo* due to dye toxicity, motion artifact and the difficulties of imaging through blood. However, there is promising small animal data using *in situ* optical mapping, which may pave the way for *in vivo* studies.^7^ Figure 1 illustrates the difference between optical mapping of AF and challenges with electrogram-based methods that we will now outline.

In patients, action potentials (activation and recovery) can be measured with monophasic action potential (MAP) catheters. Extracellular MAPs have been validated against intracellular recordings,^8^ with some discussion on their precise origin,^9^ but accurately depict action potentials and their rate dynamics in human atria and ventricles ^10,11^, alternans of APD prior to AF^12^ and spectral signatures of such traces,^13^ which assess substrate in a manner that greatly complements the limitations of electrogram only analyses However, technical difficulties in recording MAPs limit this method in humans to a small number of experienced centers.^14–18^

AF is most commonly mapped clinically using electrograms, that identify activation more than repolarization and were validated for organized rhythms such as supraventricular tachycardias^19–21^ where all sites activate in a 1:1 fashion In fibrillation, however, electrograms may show dramatic cross-talk from adjacent sites of undefined area.^11^ It is undefined if a ‘qS’ or ‘rR’ complex in AF,^22,23^ identified by rules developed for non-fibrillatory rhythms, identifies a focal beat or ‘non-local’ activation respectively - since each site integrates multiple unrelated waves of varying rate, directionality and size with no clear way of discriminating them (Figure 1).^11^

## 3. Mechanisms of Atrial Fibrillation Revealed by Optical Imaging Versus Electrogram Mapping

**Figure 2.**
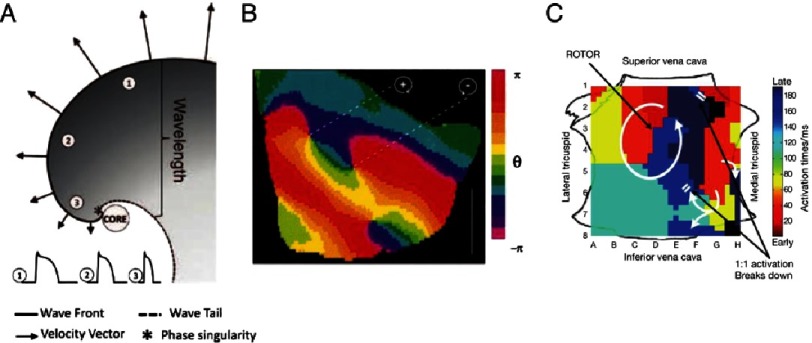
Spiral wave re-entry as drivers of cardiac fibrillation. A) Schematic of spiral wave, showing wavefront curvature as conduction velocity slows towards core (*), where wavefront meets wave-tail. This indicates the need to map activation and recovery. Action potentials from sites 1–3 show varying APD, allowing re-entry around the unexcited, yet excitable core. From [26]. B) First experimental demonstration of spiral waves in rabbit ventricular fibrillation. Phase is depicted in colour with chirality of spiral wave demarcated by +(clockwise) or –(counter-clockwise). From [2]. C) Activation map of human AF rotor showing circular activation with early-meets-late isochrones (red to blue) and fibrillatory breakdown on the periphery (double white bars). From [68].

Optical mapping studies of AF reveal that the disorder of AF is driven by localized electrical circuits in the form of spiral wave re-entry or rotors in many species^3,24,25^ including diseased human hearts.^6^ Spiral wave reentry in Figure 2A^26^ forms due to wave curvature and conduction slowing and may anchor at sites of reduced excitability such as fibrosis.^6^ From this ‘core’ (or singularity), waves emanate and breakdown to disorganized activity manifesting as fibrillation on clinical ECGs. Figure 2B shows rotors demonstrated in this way in rabbit ventricular fibrillation.

Thus, the optical mapping literature shows ‘hierarchical’ AF mechanisms between a source and disorganization in most preparations and models.^27^ Optical mapping also shows transmural differences between endo- and epicardium. When both surfaces are mapped simultaneously, rotors on one surface appear as focal activity on the other.^3,24,25,28^ Most recently optical mapping of human AF has revealed stable endocardial rotors that were eliminated by localized ablation, that showed epicardially as focal breakthroughs.^6^

In contrast to optical mapping, nearly all mapping using traditional electrogram rules in AF show complex disorganized activity^29,30^ without localized sources. A current subject of study is why traditional electrogram maps differ so dramatically from optical studies, including in human AF,^6^ but this may reflect the limitations of traditional electrogram rules in fibrillation,^11,31^ the fact that traditional studies mapped only small areas simultaneously (<10% atrial area) and that interventions to prove mechanism (as opposed to bystander) were rarely applied.^22^

Recent approaches to map clinical AF from electrograms has used global real-time mapping, incorporated rate-dependence of human atrial repolarization and conduction, combined with computer modelling using phase analysis to filter far-field signals (FIRM mapping).^32^ Such studies have revealed human AF rotors and focal sources (Figure 2C) that may arise in stable areas of ≈2cm^2^ in right or left atria, where ablation is able to eliminate AF.

These somewhat surprising properties have now been supported by optical mapping of human AF in right^6^ and left^33^ atria, and preliminary studies show a close correlation between FIRM and simultaneous optical maps in human AF.^34^ As described below, these novel approaches to mapping have improved ablation results in several multicenter studies and randomized trials are ongoing.

In summary, an intriguing dichotomy in AF is that the majority of studies that map action potentials optically – the gold standard – show localized sources that drive disorganization Conversely, traditional electrogram maps mostly do not – showing disorganization without sources. Thus, there is an urgent need to perform simultaneous optical and electrogram studies in human AF to explain these differences and, most importantly, to derive electrogram analyses that circumvent the limitations of traditional analyses in fibrillation.^22^

## 4. Clinical Observations in Support of Hierarchical AF Organization

One critical question that supports the primary mechanistic role of AF drivers is: how else can we explain the termination of persistent AF, with a globally remodeled substrate, by focal ablation? After anecdotal reports of serendipitous termination of AF by focal intervention,^35,36^ acute AF termination has been described systematically after focal ablation of rotors.^32,37,38^ Indeed, the multiwavelet reentry model cannot readily reconcile this observation (AF termination by targeted ablation), stable gradients in AF rate,^39^ vector,^40^ or electrogram shape.^41^ In addition, the localized source model is also supported by jumps rather than gradual increases in AF organization by ablation^42^ and the fact that more extensive ablation (that should constrain multiple wavelets) does not improve ablation success.^43–45^ Recent studies show how localized ablation of a driver can interfere with functional non-uniformities of the human atria to terminate AF to sinus rhythm.^46^

## 5. Management of Atrial Fibrillation using Novel Mapping Targets for Ablation

**Figure 3.**
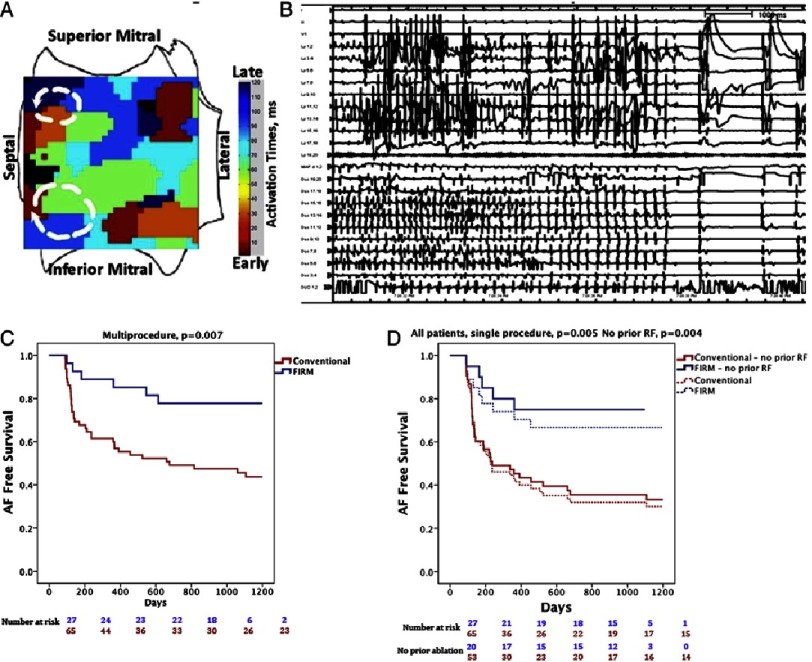
Immediate and long-term clinical success of localized ablation in human atrial fibrillation. A) Two left atrial rotors in the superior and inferior left atrial septum. Note progression of activation times (colours) as in Figure 2C. B) Abrupt termination to sinus rhythm during ablation of left inferior septal rotor during first minute of ablation in Shivkumar et al.^37^ C) Very long-term freedom from atrial fibrillation (AF) in the CONFIRM trial for FIRM-guided ablation (blue) and conventional ablation (red; P = 0.003) after 1.2 ± 0.4 procedures. D) Very long-term single-procedure freedom from the AF for FIRM-guided ablation. Data show all cases (solid lines, P = 0.002) and patients undergoing their first ablation (dashed lines, P = 0.002) From [47].

The first systematic proof of the localized source hypothesis for AF was the CONFIRM (Conventional Ablation With or Without Focal Impulse and Rotor Modulation) trial, used a novel computational mapping approach (FIRM) that interprets electrograms computationally in the context of human repolarization and phase information (Figure 3). Ninety two patients (72% persistent) were assigned prospectively to either conventional ablation or conventional plus rotor (FIRM) guided ablation.^32^ Localized sources were present in 97% of cases, each exhibiting 2–3 sources, of which one third lay in the right atrium. Brief FIRM guided ablation at each source in turn translated to higher single procedure freedom (82.4% vs. 44.9%, p < 0.001), confirmed by continuous implantable monitors in most patients, that was maintained on extended 3-year follow-up.^47^ Limitations of the CONFIRM trial include the fact that FIRM-guided ablation was performed at one center, that remapping of rotors and focal sources was not possible, and the trial was relatively small.

Several studies outside of the index center now report similar results of FIRM-guided ablation, with single-procedure freedom from AF at ≈70–80% in over 400 patients mostly with persistent AF.^48–53^ One recent study^54^ showed disappointing results lower than PV isolation success at other centers, even though PV isolation was performed As discussed by Jalife et al.,^55^ this may represent technical issues since cycle lengths in AF of 250–500ms were reported (dominant frequency 2–4 Hz, too slow for AF), or because basket catheters were inadvertently placed in left ventricle rather than the atria,^56^ or because of a challenging patient population As discussed, localized sources from a series of mapping studies including FIRM share similarities to those from optical maps of human AF

Mechanistically, other groups have had some success in identifying rotational circuits in human AF using traditional electrogram analyses.^57,58^ The fact that rotational circuits are seen at all by traditional mapping differs from prior reports showing no sources.^22^ It remains to be seen if transient rotations seen by surgical^23,59^ or body surface mapping^38^ studies reflect limitations of electrogram interpretation or rotor instability on the epicardium versus stability on the endocardium.^6^

One interesting contradiction in the body surface literature is that while activation mapping shows transient and rare rotations in AF,^60^ phase analyses show rotations consistent with sources/drivers –in the same region over prolonged periods of time where ablation can terminate AF.^38^ It is unresolved if this reflects vagaries in marking activation times (difficult in fibrillation) that is not required in phase analysis, or other factors. Technical challenges of source detection for the body surface have been identified, due to small signals, cancellation of phase singularities and amplification of drift^61^ that are relevant to this discussion. Notably, localized stable sources from phase analysis appear to agree better with optical mapping data including in humans,^6^ and explain AF termination by ablation at these sites.

**Figure 4.**
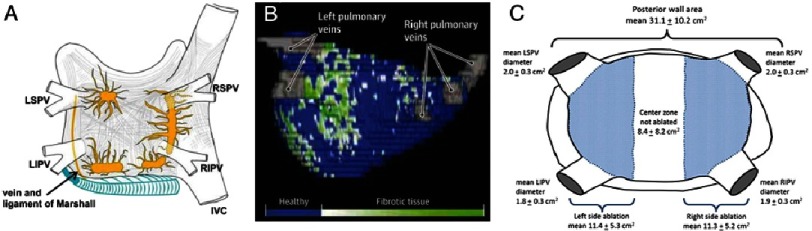
Other considerations for mechanistic ablation in AF. A) Distribution of ganglionated plexi in the left atrium, with clustering around the PVs. From [69]. B) Left atrial fibrosis imaging may inform ablation strategies. 3D left atrial shell has patchy fibrosis in a Utah Stage IV patient. From [66]. Regardless of targets, a schematic C) of left atrial area (note posterior wall alone is over 30 cm^2^) shows the extensive ablation performed (blue shading) during routine cryoballoon therapy. Note that the shaded area has significant overlap with targets in A) & B). From [67].

Overall, there is renewed attention on mechanistic targets in AF after the BOCA and STAR-AF 2 trials, which showed no benefit for widely distributed empirical ablation (stereotypical lines or complex fractionated electrograms) compared to PVI alone.^44,45^ This lack of incremental benefit with additional ablation goes against a widely distributed, non-hierarchical mechanism in human persistent AF, favoring a localized hierarchy of AF mechanisms.^62^ Other proposed mechanistic targets include ganglionated plexi (Figure 4A),^63^ areas with low voltage,^64^ fastest activation rates or dominant frequency^65^ and fibrosis (Figure 4B) ^66^. As we improve our understanding of the mechanistic targets, so we should also seek to improve lesion delivery. Current platforms such as cryoballoon have the advantage of treating significant amounts of substrate beyond the pulmonary veins (Figure 4C),^67^ allowing rapid and effective ablation although obscuring the exact mechanisms of benefit.

## Conclusions

Atrial fibrillation represents a complex pathophysiological interplay between dynamic and fixed mechanisms. Optical mapping, the gold standard for mapping arrhythmias, typically reveals localized sources for AF that produce disorganization, while studies using traditional electrogram analyses typically do not. Novel mapping techniques such as FIRM have recently shown rotors and focal sources that show similarities to optical mapping studies yet are difficult to explain by the multiwavelet disorder model While promising in early multicenter studies, ongoing randomized clinical trials will define their eventual role in treating AF based on mechanistic targets. It is expected that innovative mechanistic studies in the next few years, centered on optical mapping, will provide substantial insights into treatable and individualized mechanisms for the disease processes currently grouped as ‘atrial fibrillation’.

## Competing interests

Dr. Zaman: Reports no conflicts

Dr. Baykaner: Reports no conflicts

Dr. Schricker: Reports no conflicts

Dr. Krummen: Reports consulting work with Topera Inc. Additionally, his Institution has received fellowship support from Medtronic, Boston Scientific, St. Jude Medical and Biotronik.

Dr. Narayan: Dr. Narayan reports being co-inventor on intellectual property owned by the University of California and licensed to Topera Medical, Inc. Dr. Narayan holds equity in Topera. Dr. Narayan also reports having received consulting fees from the American College of Cardiology, St Jude Medical, Medtronic, Uptodate and Janssen Pharmaceuticals.

**Funding**

This work was supported in part by a British Heart Foundation Fellowship (FS/14/46/30907) and Fulbright Award to JZ, a HRS Research Fellowship to TB, an ACC-Merck fellowship to AAS, and grants from the National Institutes of Health (HL70529, HL83359, HL103800) to SMN.
